# Supplementary material for: Single-cell RNA-seq analysis reveals penaeid shrimp hemocyte subpopulations and cell differentiation process
Source: eLife. 2021 Jun 16;10:e66954. doi: 10.7554/eLife.66954 (PMC8266392; doi:10.7554/eLife.66954)
Supplement: Transparent reporting form [file elife-66954-transrepform.docx]

***eLife’s* transparent reporting form**

We encourage authors to provide detailed information *within their submission* to facilitate the interpretation and replication of experiments. Authors can upload supporting documentation to indicate the use of appropriate reporting guidelines for health-related research (see [EQUATOR Network](http://www.equator-network.org/%20)), life science research (see the [BioSharing Information Resource](https://biosharing.org/)), or the [ARRIVE guidelines](http://www.plosbiology.org/article/info:doi/10.1371/journal.pbio.1000412) for reporting work involving animal research. Where applicable, authors should refer to any relevant reporting standards documents in this form.

If you have any questions, please consult our Journal Policies and/or contact us: [editorial@elifesciences.org](mailto:editorial@elifesciences.org).

**Sample-size estimation**

- You should state whether an appropriate sample size was computed when the study was being designed
- You should state the statistical method of sample size computation and any required assumptions
- If no explicit power analysis was used, you should describe how you decided what sample (replicate) size (number) to use

Please outline where this information can be found within the submission (e.g., sections or figure legends), or explain why this information doesn’t apply to your submission:

For all the scRNA-seq analysis (Figures 2-8), we did not use any sample size computation nor power analysis. We had 3 independent biological replicates and sufficient number of cells (~2.6K) in total. This information can be found in the methods and results sections.

**Replicates**

- You should report how often each experiment was performed
- You should include a definition of biological versus technical replication
- The data obtained should be provided and sufficient information should be provided to indicate the number of independent biological and/or technical replicates
- If you encountered any outliers, you should describe how these were handled
- Criteria for exclusion/inclusion of data should be clearly stated
- High-throughput sequence data should be uploaded before submission, with a private link for reviewers provided (these are available from both GEO and ArrayExpress)

Please outline where this information can be found within the submission (e.g., sections or figure legends), or explain why this information doesn’t apply to your submission:

As indicated in methods and results sections pertaining to each figure, the scRNA-seq analysis was performed with 3 biological replicates (Figure 2A,B,C,D).

For the Monocle 3 (Figure 5C), we used cells from 3 biological replicates to avoid batch effects coming from biological replicates. This information can be found in results and methods section.

Imaging in Figure 9A-D: 4 independent biological replicates (n=4) were used. This information can be found in results and methods section.

FACS sorting analysis in Figure 9E: 4 independent biological replicates were used. This information can be found in results and methods section.

Validation of markers in Figure 9F: 4 independent biological replicates were used. This information can be found in results and methods section.

**Statistical reporting**

- Statistical analysis methods should be described and justified
- Raw data should be presented in figures whenever informative to do so (typically when N per group is less than 10)
- For each experiment, you should identify the statistical tests used, exact values of N, definitions of center, methods of multiple test correction, and dispersion and precision measures (e.g., mean, median, SD, SEM, confidence intervals; and, for the major substantive results, a measure of effect size (e.g., Pearson's r, Cohen's d)
- Report exact p-values wherever possible alongside the summary statistics and 95% confidence intervals. These should be reported for all key questions and not only when the p-value is less than 0.05.

Please outline where this information can be found within the submission (e.g., sections or figure legends), or explain why this information doesn’t apply to your submission:

Statistic analysis in Figure 9F: 4 independent biological replicates were used. Statistics of qRT-PCR were performed on R software. Significance between two hemocyte R1 and R2 was calculated by unpaired t test. The p values shown in the figures are represented by * (p<0.05). This information can be found in results and methods section.

(For large datasets, or papers with a very large number of statistical tests, you may upload a single table file with tests, Ns, etc., with reference to sections in the manuscript.)

**Group allocation**

- Indicate how samples were allocated into experimental groups (in the case of clinical studies, please specify allocation to treatment method); if randomization was used, please also state if restricted randomization was applied
- Indicate if masking was used during group allocation, data collection and/or data analysis

Please outline where this information can be found within the submission (e.g., sections or figure legends), or explain why this information doesn’t apply to your submission:

N.A.

**Additional data files (“source data”)**

- We encourage you to upload relevant additional data files, such as numerical data that are represented as a graph in a figure, or as a summary table
- Where provided, these should be in the most useful format, and they can be uploaded as “Source data” files linked to a main figure or table
- Include model definition files including the full list of parameters used
- Include code used for data analysis (e.g., R, MatLab)
- Avoid stating that data files are “available upon request”

Please indicate the figures or tables for which source data files have been provided:

All tables are provided as Supplementary files.

Source data files are provided to support the sinaplots in Figure 2, Figure 2–figure supplement 1.

Source data files are provided to support thepercentage of cell state in Figure 5.

Source data file is provided to support the bar graph in Figure 9F.
